# Evaluation of detection methods for Campylobacter infections among under-fives in Mwanza City, Tanzania

**DOI:** 10.11604/pamj.2014.19.392.4242

**Published:** 2014-12-18

**Authors:** Martha Fidelis Mushi, Laurent Paterno, Dennis Tappe, Anna Pendo Deogratius, Jeremiah Seni, Nyambura Moremi, Mariam Mwijuma Mirambo, Stephen Eliatosha Mshana

**Affiliations:** 1 Department of Microbiology/Immunology, Catholic University of Health and Allied Sciences, Mwanza, Tanzania; 2 Institute of Hygiene and Microbiology, University of Wuerzburg, Wuerzburg, Germany; 3 Department of Pediatric and Child Health, Catholic University of Health and Allied Sciences, Mwanza, Tanzania

**Keywords:** Campylobacteriosis, acute watery diarrhea, gram stain, 1% carbol fuchsin, preston agar

## Abstract

**Introduction:**

Campylobacter species are recognized as a major cause of acute gastroenteritis in humans throughout the world. The diagnosis is mainly based on stool culture. This study was done to evaluate the effectiveness of staining methods (Gram stain using 0.3% carbol fuchsin as counter stain and 1% carbol fuchsin direct stain) versus culture as the gold standard.

**Methods:**

A total of 300 children attending Bugando Medical Centre (BMC) and the Sekou Toure regional hospital with acute watery diarrhea were enrolled. Two sets of slides were prepared stained with 1% carbol fuchsin for 30 seconds first set, and the second set stained with Gram's stain using 0.3% carbol fuchsin as counter stain for five minutes. Concurrently, stool samples were inoculated on Preston Agar selective.

**Results:**

Of 300 stool specimens, 14(4.7%) showed positive culture after 48 hours of incubation and 28 (9.3%) shows typical morphology of Campylobacter species by both Gram stain and direct stain. The sensitivity of the Gram stain using 0.3% carbol fuchsin as counter stain and 1% carbol fuchsin simple stain versus culture as gold standard was 64.3%, with a specificity of 93.4%. The positive predictive value and negative predictive value were 32.1% and 98.2% respectively.

**Conclusion:**

The detection of Campylobacter by 1% carbol fuchsin is simple, inexpensive, and fast, with both a high sensitivity and specificity. Laboratories in settings with high prevalence of campylobacteriosis and/or limited resources can employ 1% carbol fuchsin direct stain in detecting campylobacter infections.

## Introduction

Campylobacter species are gram-negative rods with spiral, curved and/or gull wing shape. They are recognized as a major cause of acute gastroenteritis in humans throughout the world [1, 2]. Campylobacter species are transmitted through ingestion of contaminated poultry meat and other animal products [2]. A typical case of campylobacteriosis is characterized by diarrhea, fever and abdominal cramps [3]. Prevalence of Campylobacter species in developing countries among children with acute watery diarrhea is reported to range from 9%-39.6% [4, 5]. Diagnosis of campylobacteriosis is mainly based on stool culture using selective media [6] such as Skirrow's medium under a microaerophilic atmosphere [7–9]. This method is expensive, takes long time, and sometimes is associated with a high contamination rate of fecal normal flora making reading of plates difficult and time consuming [10]. Hence most laboratories in developing countries do not routinely performing test to detect campylobacter infections. Availability of cheap, sensitive, specific methods will assist in detecting campylobacter infections in developing countries and map its epidemiology. Alternative methods like the Gram stain (sensitivity of 60%-90%) and specificity of 99.5% [10, 11] has been used to detect Campylobacter species directly in stool samples. The possibility of using simple stains like 1% carbol fuchsin for the diagnosis of campylobacteriosis has previously been reported [6, 10, 11]. The current study was done to evaluate the effectiveness of staining methods (Gram stain using 0.3% carbol fuchsin as counter stain and 1% carbol fuchsin direct stain) versus culture as the gold standard).

## Methods

### Study design and site

This cross-section laboratory-based study was conducted between October 2012 and April 2013. Both inpatient and outpatient children aged 1 to 60 months attending the Bugando Medical Centre (BMC) and the Sekou Toure regional hospital with acute watery diarrhea were enrolled after informed consent. Sample size was determined by the use of Buderer's formula [12].

### Sample collection and processing

**Figure 1:**
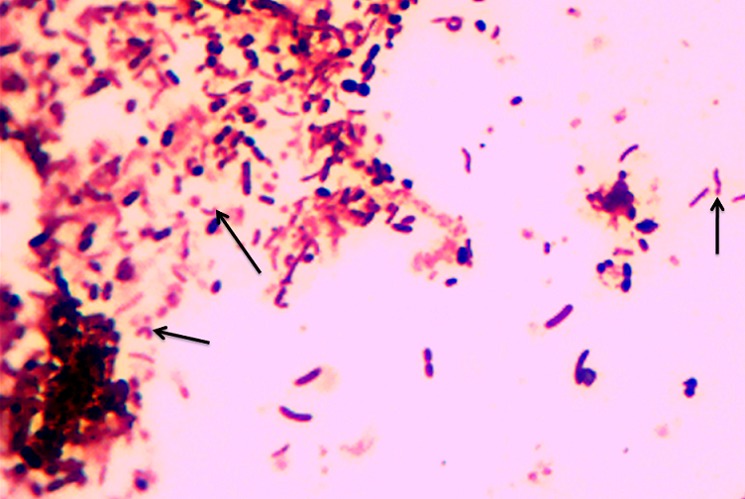
The C, S or gull wing shape of Campylobacter species (arrows) stained by 1% Carbol fuchsin. Original magnification X 100

**Figure 2:**
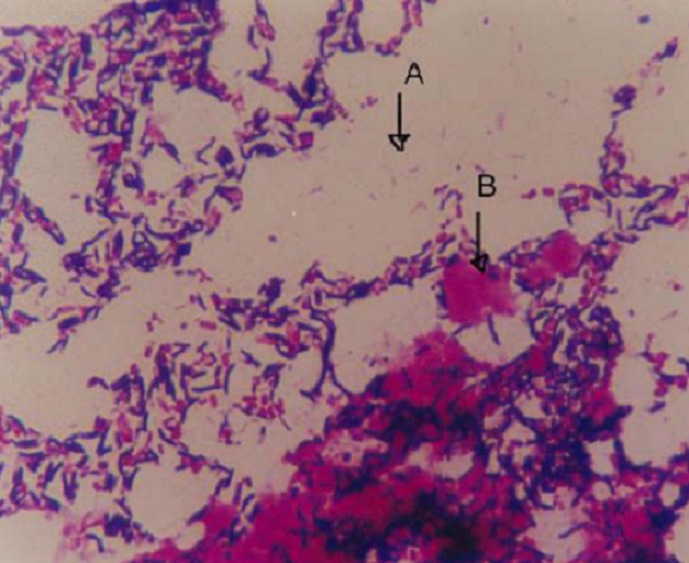
Shows the curved shape of Campylobacter specie (arrow A showing curved gram negative bacteria, arrow B showing white blood cell) stained by gram stain using 0.3% as counter stain. Original Magnification X100

Fresh stool specimens were collected using wide-mouth screw cap containers. Two slides were prepared by making thin smears, left to air dry and were subsequently heat-fixed. Staining was performed by covering the first set of smears with 1% carbol fuchsin for 30 seconds, and the second set stained with Gram′s stain using 0.3% carbol fuchsin (Sigma-Aldrich) as counter stain for five minutes. All slides were observed under light microscope using 10x magnification for white blood cells and 100x oil immersion magnification for morphological appearance of Campylobacter species (Figure 1 and Figure 2) [8, 13]. Concurrently, stool samples were inoculated on Preston Agar selective media (Oxoid, UK) (Colombia agar with lysed sheep blood and polymyxin B, trimethoprim and vancomycin). Inoculation was performed using a sterile 10 µl wire loop and media were incubated at 42oC for 48 hours under a microaerophilic atmosphere containing 5% Oxygen, 10% Carbon dioxide and 85% Nitrogen generated using a gas pack (Campy Gen,Oxoid LTD UK) [7, 9]. Suspicious colonies were further identified using oxidase and catalase testing, and Gram staining [14]. The research protocol was approved by the Joint BMC/CUHAS ethics committee (CREC/004/2013).

### Data Management and analysis

Data was analyzed using STATA version 11 software. For calculation of sensitivity and specificity, predictive values, positive and negative likelihood ratios, two by two tables were used and for association the Chi square test was used. For quality control Campylobacter jejuni ATCC 700819 strain was used for positive control during incubation whereas the Pseudomonas aeruginosa ATCC 9027 strain and Staphylococcus aureus ATCC 25923 strains were used as a positive control for oxidase and catalase testing, respectively. Smear and culture results were blinded. All slides were examined by two individuals independently with a light microscope using x10 magnification for white blood cells and x100 oil immersion magnification for the presence of Campylobacter species. Positive results were verified by different microbiologists before culture results were known.

## Results

**Figure 3:**
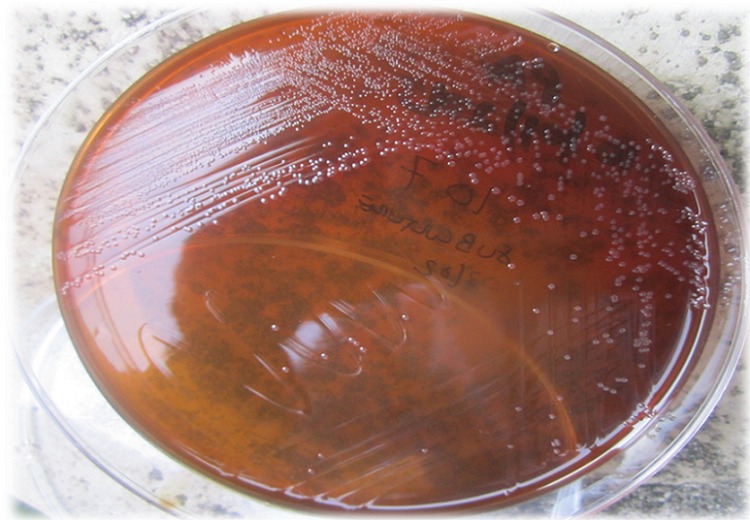
Colonies of Campylobacter species grown on Preston Agar, showing translucent droplet-like appearance

**Figure 4:**
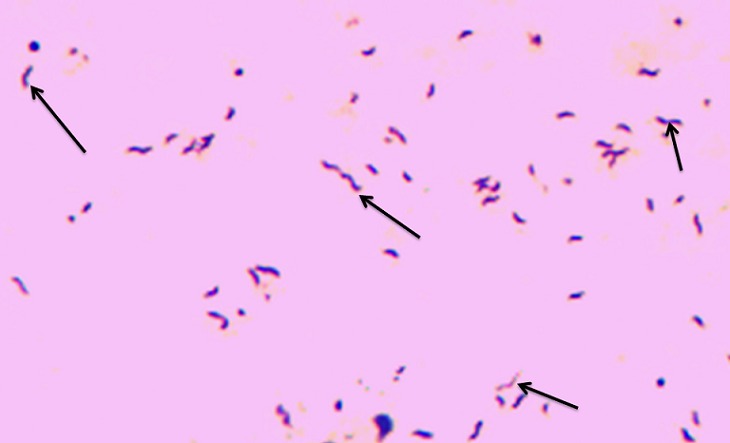
Shows Cells of Campylobacter species from isolate colony showing recognized “comma” or “gull wing”(arrows) shape stained with Gram stain. Original magnification X 100

During the study period 1787 and 2275 children were admitted to BMC and Sekou Toure hospital, respectively. A total of 300 children with a median age of 12 (Range, 8-19) months meet the study criteria and were enrolled. Of these, 169 (56.5%) were enrolled from BMC and 131 (43.7%) were enrolled from Sekou Toure hospital. The majority of children were males 170 (56.7%). Of the enrolled children, 205 (68.3%) had a temperature above 37.5^o^ C. Three hundred stool specimens were investigated for campylobacter infection. Most of the samples analyzed were mucoid (71.0%), (Table 1). Of 300 stool specimen cultured, 14 (4.7%) showed positive after 48 hours of incubation. Translucent drop-like colonies on the surface of the agar plate (Figure 3) was the commonest feature observed for culture-positive samples. Gram stain was repeated for culture positive isolates (Figure 4). While of 300 stool specimen 28 (9.3%) shows typical morphology of Campylobacter species by both Gram stain using 0.3% carbol fuchsin and direct stain using 1% Carbol fuchsin (Table 2). The sensitivity of the Gram stain using 0.3% carbol fuchsin as counter stain and 1% carbol fuchsin simple stain versus culture as reference standard was 64.4%, with a specificity of 93.4%. The positive predictive value was 32.1%, the negative predictive value was 98.2%; this resulted in a positive likelihood ratio of 9.1, a negative likelihood ratio of 0.4, and an accuracy of 92% (Table 2). The association of presence of white blood cells in stool and Campylobacter species infection was investigated as shown in Table 3. Of 33 stool specimens with WBC, 26 (78.8%) had campylobacter infection while of 267 stool specimens with no WBC only 7 (2.6%) had campylobacter infection (p

**Table 1:** Macroscopic appearance of stool specimens

Appearance of stool	Number	Percent
Watery	10	3.3
Semi formed	77	25.7
Mucoid	213	71.0
Total	300	100

**Table 2:** Microscopic (Gram stain using 0.3% carbol fuchsin as counter stain and 1% carbol fuchsin) versus culture as reference standard

Microscopic	Culture		Total
	Negative	Positive	
Negative	267 (93.4%)	5 (35.7%)	272 (90.7%)
Positive	19 (6.6%)	9 (64.4%)	28 (9.3%)
Total	286(100%)	14 (100%)	300 (100%)

Positive predictive value was 9/28 (32.1%), Negative predictive value 267/272 (98.2%), Positive likelyhood ratio= 64.4/1-93.4 (9.1)

Negative likelyhood ratio= 1-0.64/0.93 (0.4)

**Table 3:** Association between presence of WBC and *Campylobacter species* in stool

WBC	Presence of *Campylobacter species*		Total
	Negative	Positive	
No	260 (97.4%)	7 (2.6%)	267 (100%)
Yes	7 (21.2%)	26 (78.8%)	33 (100%)
Total	267 (89.0%)	33 (11%)	300 (100%)

The presence of white blood cells in stool samples is significantly associated with a campylobacter infection (p <0.001).

## Discussion

Gram stain has been used as principle stain in most laboratories in developing countries as a critical step in the diagnosis of bacterial infections. It's useful administration in visualization of Campylobacter species using 0.3% carbol fuchsin as counter stain for five minutes has been documented [10]. In the current study, Gram staining for the examination of stool for Campylobacter specie had a sensitivity of 64.3% and a specificity of 93.4%. These data correspond to the findings of other studies, in which the sensitivity has been shown to be 60%-94% [10–16]. Using the Gram stain, examination for fecal white blood cells as sign of infection can be performed concurrently with examination for the presence of campylobacteriosis in stool. In the current study the presence of white blood cells in stool was detected in 78.8% of positive samples. This was statistically significantly associated with a campylobacter infection (p 8, 17, 18], can be used as good predictor of mucosa-damaging intestinal infections, such as campylobacteriosis [10]. In previous studies the occurrence of white blood cells in stool has been reported in 25%-90.4% of culture-positive cases of Campylobacter species infections [10, 16]. However, other tissue-damaging bacterial infections of the intestines, such as salmonellosis, shigellosis, also lead to the presence of white blood cells in stool. A simple stain like 1% carbol fuchsin is cheap and the examination has a short turn-around time. Thus, 5-10 minutes after the stool sample has been sent to the laboratory, the clinician might have the results for the test already. In the current study we evaluated the performance of a 1% carbol fuchsin simple stain versus culture as reference standard and its sensitivity and specificity was similar to that of the Gram stain (64.3% and 93.4% versus 64.4% and 93.4%). However, Gram staining usually showed Gram-negative curved rods which stained only faintly (Figure 2 and Figure 3), making detection by inexperienced personnel difficult, thus lowering the sensitivity of the technique [19]. In contrast, using 1% carbol fuchsin displays the recognized shapes of Campylobacter species cells very clearly and makes this stain superior to the conventional Gram stain (Figure 1). It has been documented that morphology of cells on solid media can change over time in older cultures from spiral to coccoid forms, leading to false-negative results [20].

Stool culture for the detection of Campylobacter species using selective media is expensive, often an unavailable and time consuming technique [6]. In this study culture was used as reference standard for diagnosis of enteric campylobacteriosis. We detected 14 (4.7%) culture-positive samples based on their growth morphology (round, convex, translucent droplet/colourless-like colonies [8, 9]. In this study, 19 samples were smear-positive but turned out to be negative in culture. This could be due to the previous use of antibiotics, poor survival of this organism during laboratory culturing techniques [10]. These samples were traced, 9 patients had used antibiotics while 10 had not used antibiotic prior to study. For the other 10 samples which were smear-positive but turned out to be negative in culture could be due to poor survival on bench or microscopic error or some strains becoming susceptible to the antibiotics in the media [10, 21, 22]. Five samples were smear negative but culture positive these samples were considered as false negative. Low shading of these bacteria due to use of antibiotic or late stage of the infection can explain this.

## Conclusion

The detection of Campylobacter species by 1% carbol fuchsin is simple, inexpensive, and fast, with both a high sensitivity and specificity. Laboratories in settings with high prevalence of campylobacteriosis and/or limited resources can mull over using direct stain by simple stain 1% carbol fuchsin in detection of campylobacter infections.
